# CpG-induced antitumor immunity requires IL-12 in expansion of effector cells and down-regulation of PD-1

**DOI:** 10.18632/oncotarget.11833

**Published:** 2016-09-02

**Authors:** Peng Yin, Xin Liu, Aaron S. Mansfield, Susan M. Harrington, Yinghua Li, Yiyi Yan, Haidong Dong

**Affiliations:** ^1^ Department of Urology, Mayo Clinic, Rochester, MN, USA; ^2^ Department of Immunology, Mayo Clinic, Rochester, MN, USA; ^3^ Division of Medical Oncology, Mayo Clinic, Rochester, MN, USA

**Keywords:** CpG ODN, IL-12, immune checkpoints, PD-1, tumor immunotherapy

## Abstract

CpG oligodeoxynucleotides, as a ligand of toll-like receptor (TLR)-9, have demonstrated promising antitumor effects in some clinical trials; however, its toxicity and low efficacy as a systemic therapy has limited its therapeutic applications. In order to improve its therapeutic efficacy, we investigated the mechanisms of CpG-induced antitumor immunity in the context of CD8^+^ T cell responses. We show that IL-12 is required for the expansion of IFN-γ producing tumor-reactive CD8^+^ T cells capable of rejecting tumors. In addition, CpGs reduced PD-1 expression by effector CD8^+^ T cells via the IL-12 pathway. The combination of CpG and PD-1 blockade show a synergistic effect in generation of systemic antitumor immunity. Our studies define a critical role of IL-12 in CpG-induced antitumor immunity and provide a rationale for combined therapy with TLR agonists and immune checkpoint blockade in cancer treatment.

## INTRODUCTION

CpG oligodeoxynucleotides (ODN) are DNAs containing unmethylated deoxycytidylyl-deoxyguanosine dinucleotides. Since the unmethylated CG sequence is characteristic of bacterial DNA and injection of dead bacteria occasionally shows antitumor effects in humans [1], CpGs have been explored as an immune adjuvant in the treatment of human cancers [2, 3]. Although modest activity has been observed for CpG treatment in several clinical trials [4], frequent adverse events and low efficacy led to the early termination of these trials. In order to improve therapeutic outcomes and to reduce the adverse effects of CpGs, further exploration of the mechanism of function of CpGs is required.

Through its recognition by Toll-like receptor (TLR)-9 [5], CpGs induce the production of cytokines from dendritic cells, including IL-12 [6], and initiate a cascade of innate and adaptive immune responses to tumors [7]. It has been reported that intratumoral injection of CpGs stimulates the production of IL-12 and other Th1 cytokines that promote antitumor immunity [8]. But to what degree IL-12 is required by CpG in the induction of antitumor immunity is not clear. The influence of IL-12 on CpG-induced antitumor CD8^+^ T cell responses is not fully understood.

Endogenous antitumor CD8^+^ T cell responses have been observed in established tumor tissues [9, 10], their accumulation and function are tightly controlled by immune suppressor cells and immune checkpoint molecules expressed by tumors and stromal cells. Even though PD-1 blockade enhances tumor eradication with CpG therapy [11], it is unclear how PD-1 influences the antitumor T cell responses induced by CpGs and whether PD-1 expression is regulated by CpG treatment.

In this study, we examined the role of IL-12 in the antitumor function of CpGs using IL-12 knockout mice. We found that IL-12 was required for CpGs to expand IFN-γ producing tumor-reactive CD8^+^ T cells and to down-regulate PD-1 expression by tumor–reactive CD8^+^ T cells. Importantly, the combination of CpG and PD-1 blockade show a synergistic effect in generation of a systemic antitumor immunity. Our studies define a critical role of IL-12 in CpG-induced antitumor immunity and provide a rationale for combined therapy with TLR agonists and immune checkpoint blockade in cancer treatment.

## RESULTS

### Antitumor function of CpG is Interleukin-12 (IL-12) dependent

**Figure 1:**
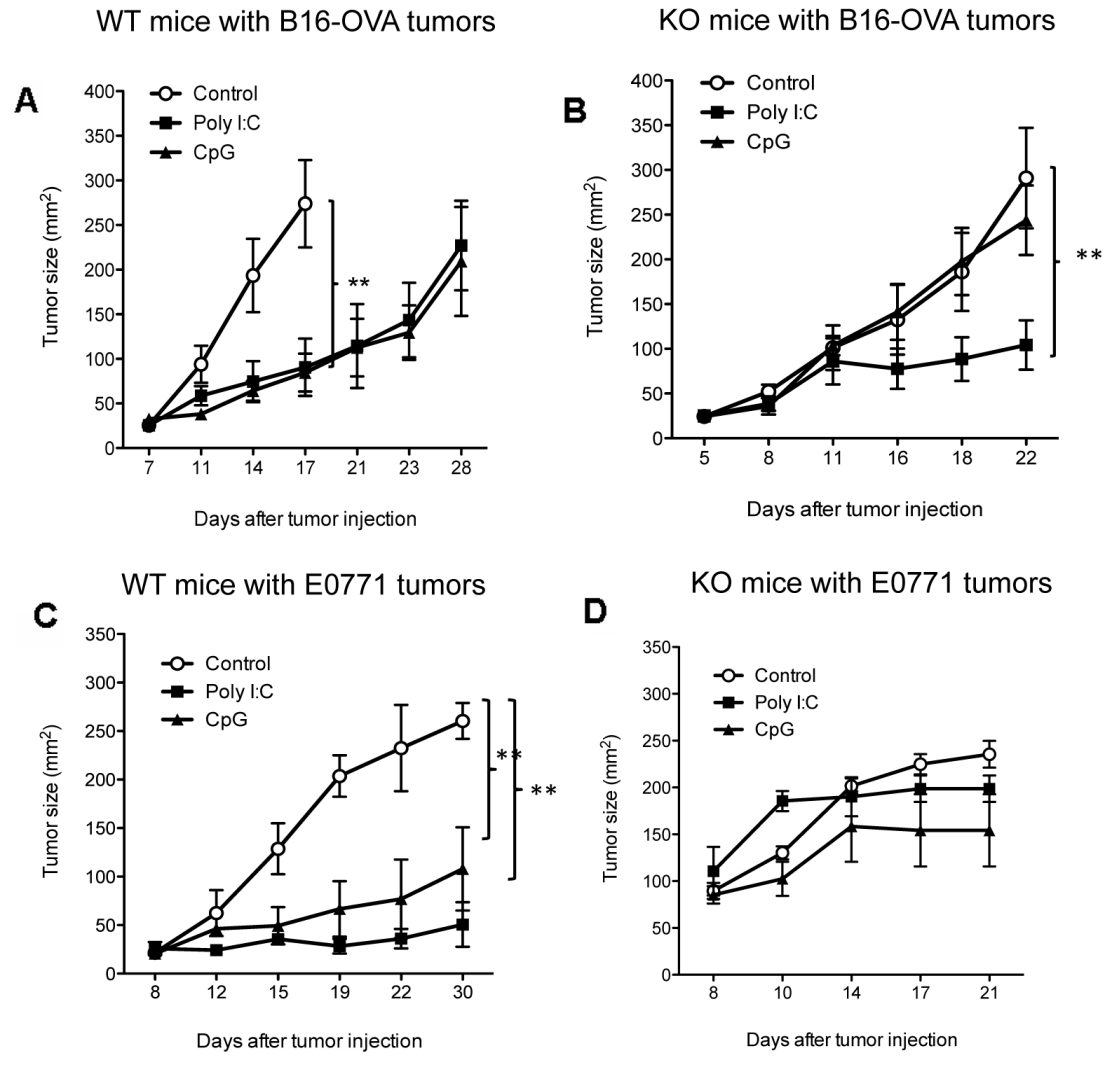
IL-12 is required in antitumor function of CpG Tumor cells were subcutaneously injected into WT-B6 (**A.** B16-OVA; **C.** E0771) and IL-12-deficient (KO) (**B.** B16-OVA; **D.** E0771) mice. On day 7 after tumor injection, CpG, Poly I:C, or control (carrier PBS) were injected into tumor tissues (i.t.) daily for three doses. Data show mean ± s.e.m. of 5 mice per group of WT mice and IL-12 KO mice. **P< 0.01 between control and CpG or Poly I:C groups (Two-way ANOVA).

To understand the role of IL-12 in antitumor immunity induced by TLR ligands, we compared the antitumor function of the TLR9 ligand CpG and TLR3 ligand Poly I:C among wild type (WT) mice and IL-12 deficient (KO) mice. Once B16-OVA tumors established in WT or IL-12 KO mice, we performed intratumoral injections of CpG, Poly I:C, or PBS (carrier control) daily (on days 7-9 post tumor injection) for a total of three doses. In WT mice, both CpG and Poly I:C demonstrated significant antitumor function by suppressing tumor growth in comparison with no-treatment controls (PBS only) (Figure 1A). However, the antitumor function of CpG, but not Poly I:C, was not observed in IL-12 KO mice (Figure 1B). In addition to the murine melanoma (B16-OVA) tumor model, we tested the antitumor function of CpG in another tumor model E0771, a murine breast cancer. Both CpG and Poly I:C significantly suppressed the growth of E0771 tumors in WT mice (Figure 1C) but not in IL-12 KO mice (Figure 1D). Our results suggest that IL-12 is required in CpG-induced antitumor immunity.

### IL-12 is required for CpGs to expand effector CD8^+^ T cells within tumors

**Figure 2:**
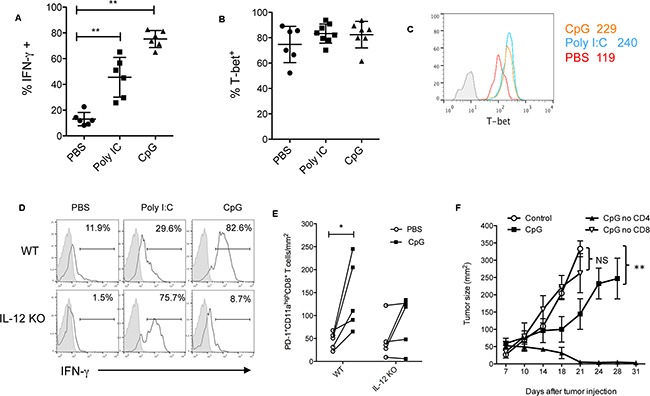
CpG-induced effector CD8+ T cell responses are dependent on IL-12 Frequency of IFN-γ^+^ **A.** and T-bet **B.** among tumor-reactive PD-1^+^ CD11a^high^ CD8^+^ T cells isolated from tumor tissues on day 2 after least treatments of PBS, CpG or Poly I:C. Data shows mean ± s.d. of 6 mice per group. **P<0.01 between PBS and CpG or Poly I:C groups (Two-tailed Mann-Whitney test). A histogram shows the levels of T-bet in tumor reactive CD8^+^ T cells **C.** Numbers are MFI (mean fluorescence intensity) of T-bet. The isotype control for T-bet staining is shown in filled gray lines (MFI: 8). **D.** Reduced frequency of IFN-γ^+^ cells in tumor-reactive CD8^+^ T cells in IL-12 KO mice compared with WT mice. **E.** Quantification of tumor-reactive CD11a^high^PD-1^+^ CD8^+^ T cells within tumor tissues following CpG treatment. Data are numbers of cells per mm^2^ of tumor size. **F.** Depletion of CD8^+^ T cells, but not CD4^+^ T cells, abolished the antitumor effects of CpG treatment. *P<0.05, **P< 0.01 compared with control (PBS)-treated groups (Two-way ANOVA). NS: non-significant.

To understand how IL-12 contributes to CpG-mediated antitumor immunity, we measured and compared the frequency of IFN-γ producing tumor-reactive CD8^+^ T cells in tumor tissues following treatments. PD-1^+^CD11a^high^ expression by CD8^+^ T cells was used as a surrogate marker to identify tumor-reactive CD8^+^ T cells [9, 12]. CD8^+^ T cells were isolated from tumor tissues two days after last treatment. After a brief re-stimulation, PD-1^+^CD11a^high^ CD8^+^ T cells were analyzed for intracellular production of IFN-γ. Both CpG and Poly I:C increased the frequency of IFN-γ producing tumor-reactive CD8^+^ T cells compared with PBS control in WT mice (Figure 2A). Since most tumor-reactive CD8^+^ T cells expressed T-bet, a transcription factor for Th1 or effector CD8^+^ T cells [13], we examined whether the treatment with CpG or Poly I:C would increase the frequency of T-bet^+^ CD8^+^ T cells. The results of Figure 2B show that the frequency of T-bet^+^ CD8^+^ T cells did not dramatically increase upon treatment with CpG or Poly I:C. However, both CpG and Poly I:C increased T-bet levels (MFI) in tumor-reactive (PD-1^+^CD11a^high^ CD8^+^) T cells (Figure 2C). In IL-12 KO mice, CpG was not able to increase the frequency of IFN-γ producing tumor-reactive CD8^+^ T cells compared with PBS control, while Poly I:C maintained its ability to do so (Figure 2D). In addition to their frequency, the absolute numbers of tumor-reactive CD8^+^ T cells increased in tumors treated with CpG (Figure 2E).

To determine what effector T cell subsets are required in CpG-induced antitumor immunity, we depleted CD4 or CD8 T cells before CpG treatment. We found a depletion of CD8^+^ T cells, but not CD4^+^ T cells, significantly abolished the antitumor function of CpGs (Figure 2F), suggesting CD8^+^ T cells are required for CpG-mediated antitumor immunity. Of note, although CD4^+^ T cells are not required in this setting (Figure 2F), the depletion of CD4^+^ T cells enhanced the therapeutic effects of CpGs probably via depletion of CD4^+^ Treg cells that limit CpG's antitumor activity [14]. Taken together, our results suggest that IL-12 is required for CpG to expand effector CD8^+^ T cells capable of rejecting tumors.

### IL-12 is required for CpG-induced down-regulation of PD-1 in CD8^+^ T cells

**Figure 3:**
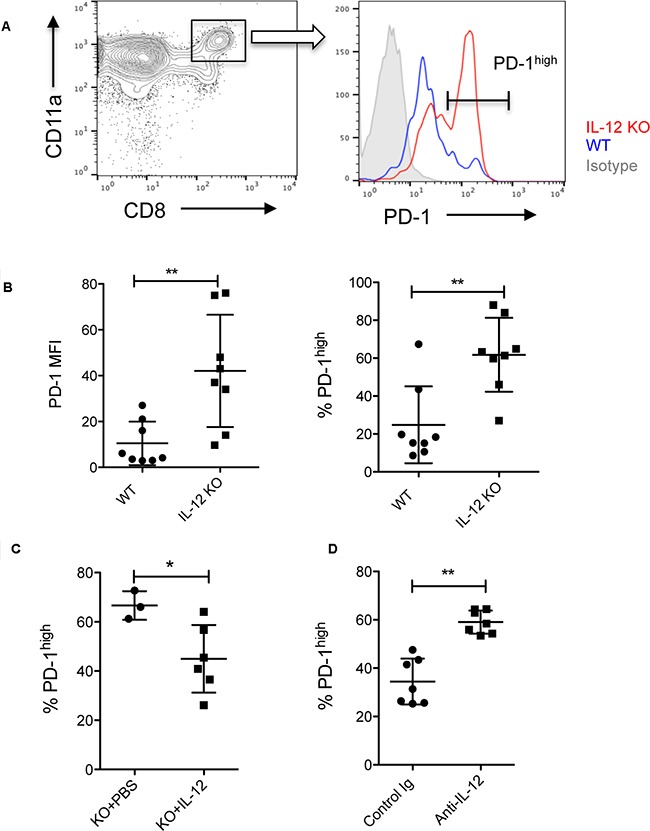
IL-12 reduces PD-1 expression by tumor-reactive CD8+ T cells **A.** PD-1 levels were measured in tumor-reactive CD11a^high^ CD8^+^ T cells within tumor tissues on day 11 after tumor (B16-OVA) injection in WT and IL-12 KO mice. A histogram shows higher PD-1 expression in IL-12 KO mice than in WT mice. Negative control of PD-1 staining is shown in filled gray lines. **B.** Both frequency and MFI (mean fluorescent intensity) of PD-1 expression were higher in tumor-reactive CD8^+^ T cells in IL-12 KO mice than in WT mice. PD-1 expression by tumor-reactive CD8+ T cells within tumors after intratumoral injection of mouse IL-12 **C.** or anti-mouse IL-12 neutralizing antibody **D.** Data show mean ± s.d. of 5-8 mice per group. *P<0.05, **P<0.01 between WT and IL-12 KO mice, IL-12 or anti-IL-12 treated and non-treated mice (Two-tailed Mann-Whitney test).

To understand the mechanism behind the loss of CpG's antitumor function in IL-12 KO mice, we compared the profile of tumor-reactive CD8^+^ T cells between WT mice and IL-12 KO mice. Eleven days after tumor injection, we found the frequency of PD-1^high^ cells among CD11a^high^ CD8^+^ T cell population significantly increased in IL-12 KO mice compared with WT mice (Figure 3A and 3B). The actual levels of PD-1 (based their MFI) were also elevated in CD11a^high^ CD8^+^ T cells isolated from tumor tissues in IL-12 KO mice (Figure 3B). To test whether addition of IL-12 back to IL-12 KO mice would reduce PD-1 expression, we injected IL-12 into tumor tissues in IL-12 KO mice on days of 8 to 10. The results of Figure 3C show that IL-12 significantly decreased the frequency of PD-1^high^ cell among CD11a^high^ CD8^+^ T cells within tumors. On the other hand, in WT mice the injection of anti-IL-12 neutralizing antibody dramatically increased the frequency of PD-1^high^ cells among CD11a^high^ CD8^+^ T cells within tumors (Figure 3D). Collectively, our results suggest that IL-12 is required in down-regulation of PD-1 expression by tumor-reactive CD8^+^T cells.

**Figure 4:**
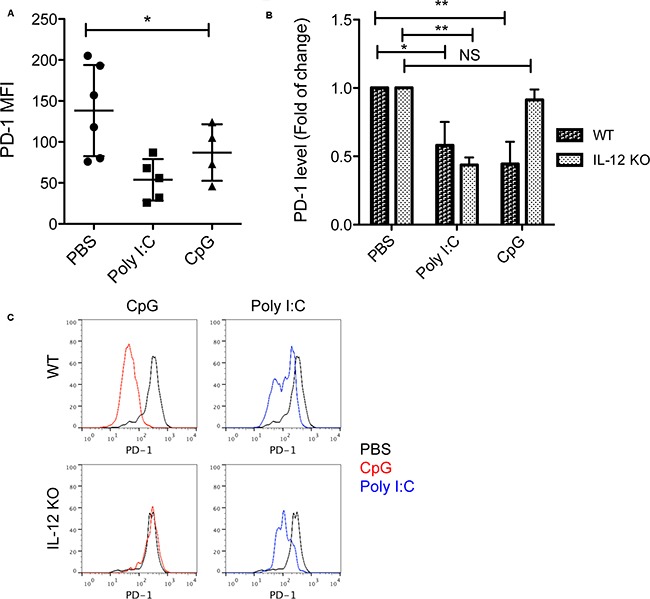
Down-regulation of PD-1 induced by CpG is dependent on IL-12 **A.** PD-1 levels (MFI) among tumor-reactive CD11a^high^ CD8^+^ T cells isolated from tumors one day after last treatments with CpG, Poly I:C or PBS. Data show mean ± s.d. of 4-6 mice per group, *P<0.05 compared with PBS groups (One-way ANOVA). **B.** Fold of Changes of PD-1 levels in tumor-reactive CD8^+^ T cells isolated from tumors treated with CpG or Poly I:C in WT mice and IL-12 KO mice (n=4). *P<0.05, **P<0.01 compared with PBS groups in WT or IL-12 KO mice (One-way ANOVA). NS: non-significant. **C.** Representative histograms show PD-1 expression by CD11a^high^ CD8^+^ T cells in tumor tissues following treatment with CpG or Poly I:C in WT or IL-12 KO mice. Black lines show the baseline levels of PD-1 in PBS treated groups.

To test whether CpGs suppress PD-1 expression in a IL-12 dependent way, we measured PD-1 levels in tumor-reactive CD11a^high^ CD8^+^ T cells isolated from tumor tissues following treatment with CpG, Poly I:C or PBS in WT and IL-12 KO mice. Both CpG and Poly I:C treatments reduced the levels of PD-1 among CD11a^high^ CD8^+^ T cells within tumors in WT mice (Figure 4A). However, CpG treatment was not able to reduce PD-1 expression in IL-12 KO mice, suggesting that the down-regulation of PD-1 induced by CpGs is dependent on IL-12 (Figure 4B). In contrast to CpG, Poly I:C induced a similar degree of reduction of PD-1 in both WT and IL-12 KO mice (Figure 4B). Since PD-1 plays a key role in limiting endogenous T cell responses to tumors [9, 15], our results suggest that down-regulation of PD-1 is a new function of CpG in promoting antitumor CD8^+^ T cell responses.

### A synergistic effect of CpG and PD-1 blockade in treatment of established tumors

**Figure 5:**
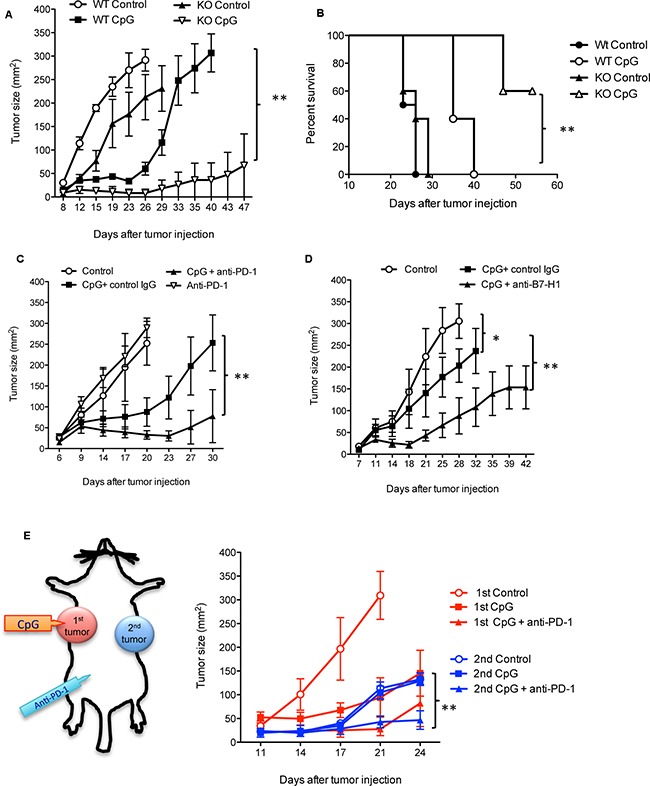
A synergistic effect of CpG and PD-1 blockade in treatment of established tumors Tumor (B16-OVA) growth **A.** and survival of animals **B.** in WT mice and PD-1 KO mice treated with CpG or PBS (Control). Data show mean ± s.e.m. of 5 mice per group of WT mice and PD-1 KO mice. **P<0.01 between WT CpG and PD-1KO CpG groups (Two-way ANONVA). The survival curves were generated by Kaplan-Meier method, ** P<0.01 between WT CpG and PD-1KO CpG groups (Gehan-Wilcoxon test). **C.** Tumor growth in WT mice treated with PBS (control), CpG plus control IgG, anti-PD-1, or CpG plus anti-PD-1. **D.** Tumor growth in WT mice treated PBS (control), CpG plus control IgG or CpG plus anti-B7-H1. Data show mean ± s.e.m. of 5 mice per group. *P<0.05 between control groups and CpG plus control Ig groups, **P<0.01 between CpG plus control Ig vs. CpG plus anti-PD-1 or anti-B7-H1 (Two-way ANONVA). **E.** The 1st tumors were injected at the right flanks. The 2^nd^ tumors were injected 2 days later at the left flanks in the same mice. Only the 1^st^ tumors received intratumoral injection of CpG or PBS (control) followed with or without i.p. injection of anti-PD-1 antibody. Data show mean ± s.e.m. of three mice per group. **P<0.01 between control vs. CpG plus anti-PD-1 (Two-way ANONVA).

Since both CpG and PD-1 blockade target PD-1 expression or PD-1 function, we then tested whether there is a synergistic effect of CpG and PD-1 blockade in treatment of established tumors. We first examined the therapeutic effects of CpGs in mice with genetical defects in PD-1 expression. CpG treatment significantly suppressed tumor growth in PD-1 KO mice compared to WT mice (Figure 5A). The survival of CpG-treated mice lived longer in PD-1 KO mice than in WT mice (Figure 5B). We then evaluated the therapeutic effects of the combination of CpGs with blocking antibody to PD-1 or B7-H1 in WT mice. We found that combined therapy significantly suppressed tumor growth compared to either therapy when used alone (Figure 5C–5D). To test whether the combined therapy would generate systemic antitumor immunity, we injected B16-OVA tumor cells in the same mice at two different sites (right and left flanks). Only the first tumors at right flanks were injected with CpG followed with or without i.p. injection of anti-PD-1 antibody (Figure 5E). The growth of the 2^nd^ tumors was suppressed in mice when the 1^st^ tumors were injected with CpG followed with anti-PD-1 treatment, while CpG injection in 1^st^ tumors alone did not suppress the 2^nd^ tumor growth (Figure 5E). Our results suggest that intratumoral injection of CpG induces a local antitumor immunity that can be enhanced by PD-1 blockade to achieve systemic protection.

## DISCUSSION

In this study, we defined a critical role of IL-12 in CpG-induced antitumor immunity. IL-12 is required at least in two aspects regarding to tumor-reactive CD8^+^ T cell responses. First, IL-12 is required in the expansion of IFN-γ producing effector CD8+ T cells within tumors (Figure 2). Second, IL-12 is required in down-regulation of PD-1, a key immune checkpoint molecule that limits the effector function of antitumor CD8^+^ T cells (Figure 3–4). We also found that local antitumor immunity induced by CpG can be enhanced by PD-1 blockade to provide a systemic immunity against tumors.

Since we administered CpG directly into tumor tissues, CpG injection may impact tumor-reactive CD8^+^ T cell responses locally and systemically. We previously reported that antigen stimulation leads to prompt up-regulation of PD-1 in primed CD8^+^ T cells. If PD-1 is blocked, more primed CD8^+^ T cells will become effector cells [16], an outcome similar to that induced by IL-12 when it was included at this stage of T cell priming [17]. In this scenario, down-regulation of PD-1 induced by CpG may contribute to the expansion of effector CD8^+^ T cells at the primary tumor sites and suppresses tumor growth (Figure 2E and 5E). The expanded PD-1^+^ effector CD8^+^ T cells may circulate in blood and migrate to the secondary tumor sites, but their antitumor function may be inhibited by B7-H1 expressing tumors [18, 19]. Therefore, CpG injection at the 1^st^ tumors did not suppress the growth of the 2^nd^ tumors that can only be suppressed when CpG injection is combined with PD-1 blockade (Figure 5E). Thus, to enhance the therapeutic effects of CpG, PD-1 blockade is required to maintain a durable and systemic antitumor CD8^+^ T cell responses.

The potential down-regulation of PD-1 is a new function of CpG through IL-12 pathway. Some in vitro studies imply that IL-12 regulates PD-1 expression by primed CD8^+^ T cells [17, 20]. Schurich et al observed that human viral-specific CD8^+^ T cells expressed lower levels of PD-1 in culture with IL-12 than in culture without IL-12 [20]. However, it is not clear whether IL-12 has the same role in vivo. Using IL-12 KO mice, we observed an increased PD-1 expression by tumor-reactive CD8^+^ T cells in the absence of IL-12 in vivo (Figure 3) and this increase of PD-1 can be reverted by addition of exogenous IL-12 (Figure 3C). In addition, when IL-12 is blocked in WT mice by anti-IL-12 neutralizing antibody, PD-1 expression increased in tumor-reactive CD8^+^ T cells (Figure 3D). Our results define a key role of IL-12 in the regulation PD-1 expression in antigen-primed CD8^+^ T cells. It is likely that CpG/IL-12 does so via up-regulation of T-bet. T-bet has a potential role in repression of PD-1 expression by binding to the regulatory region upstream of *PDCD1*, the gene that encodes PD-1 [21]. Although CpG treatment did not increase the frequency of T-bet^+^ in CD8^+^ T cells, the T-bet levels were increased in tumor-reactive CD8^+^ T cells by CpG (Figure 2C), suggesting that CpG signals increase the expression of T-bet at a single cell level that may contribute to PD-1 down-regulation.

The dependency on IL-12 seems to be a unique feature of CpG as IL-12 deficiency did not affect Poly I:C-induced PD-1 down-regulation and antitumor activity (Figure 1 and 2). At least in this respect, Poly I:C was superior to CpG when tumor-bearing hosts have compromised IL-12 production. As summarized in a recent review, the combination of poly I:C and PD-1 blockade has emerged as a promising therapy for cancer in several preclinical models [22]. However, the potential function of poly I:C in up-regulating immune checkpoint molecules in antigen-presenting cells [23] and the lack of TLR3 in human plasmacytoid dendritic cells [24] may limit the use of poly I:C for treating tumors in humans. Thus, optimizing the selection or combination of TLR ligands to maximize their therapeutic effects warrants further investigation.

Since PD-1 blockade has been approved by the FDA to treat metastatic melanoma, non-small cell lung cancer and kidney cancers, our study provides a novel approach to improve the efficacy of PD-1 or B7-H1 blockade therapy by including CpG. Recently, the role of gut microbiota has been addressed in improving immune checkpoint blockade therapy [25, 26]. In these studies, the hosts that harbor specific bacterial species (*Bifidobactrium or Bacteroidales*) demonstrate increased responses to anti-PD-L1 or anti-CTLA-4 therapy [25, 26]. Importantly, bacteria (i.e. *Bifidobactrium*) alter dendritic cell activity that eventually leads to enhanced function of tumor-reactive CD8^+^ T cells [25]. It would be interesting to know whether these specific bacterial species are rich in CpG DNA and/or are actively releasing CpG DNA that activates DC function in vivo. Thus, our studies add novel knowledge in respect to the mechanisms by which the components of bacteria regulating antitumor immunity via IL-12 and PD-1 expression by immune cells.

In summary, our study defined a critical role of IL-12 in CpG-induced antitumor immunity. IL-12 is required for CpG to expand tumor-reactive CD8^+^ T cells capable of rejecting tumors and to reduce PD-1 expression by effector CD8^+^ T cells. Importantly, the combination of CpG and PD-1 blockade show a synergistic effect in generation of systemic antitumor immunity. Our results provide a rationale for future clinical trials of combination therapy of TLR agonists and immune checkpoint blockade in treatment of advanced human cancers.

## MATERIALS AND METHODS

### Mice

Eight to twelve week-old C57BL/6 wild type or IL-12 knockout mice were obtained from Taconic Animal Laboratory (Germantown, NY) or Jackson Lab (Bar Harbor, ME) and maintained under pathogen-free conditions in the animal facility at Mayo Clinic's Comparative Medicine Department. PD-1 KO C57BL/6 mice were provided by L. Chen (Yale University, New Haven, CT) with the permission of Dr. T. Honjo (Kyoto University). All animal experiments were performed according to the protocols approved by the Institutional Animal Care and Use Committee of Mayo Clinic.

### Cell lines and reagents

B16-OVA and E0771 are murine melanoma and breast cancer cell lines, respectively, from a C57BL/6 background, and were obtained from Dr. Vile and Dr. Schrum (Mayo Clinic). Both B16-OVA and E0771 cells underwent quality control analysis by reverse PCR in Drs. Vile and Schrum's lab prior to receipt. B16-OVA cells and E0771 were passaged for less than six months in the described experiments. All cell lines were free of Mycoplasma contamination (Mycoplasma Detection kit; Roche Diagnostics, Chicago, IL). Tumor cells were cultured in DMEM (Invitrogen Corporation, Carlsbad, CA) or RPMI 1640 medium (Cellgro, Hendon, VA) with 10-20% FBS (Life Technologies, Carlsbad, CA), 1 U/ml penicillin, 1 μg/ml streptomycin, and 20 mM HEPES buffer (all from Mediatech, Manassas, VA). Anti-PD-1 hamster mAb G4 or anti-B7-H1 (aka PD-L1) hamster mAb 10B5 were purified as previously described [15]. CpG ODN- 1668 and Poly I:C were purchased from InvivoGen (San Diego, CA).

### Tumor challenge and treatment

C57BL/6 WT mice or IL-12 KO mice in C57BL/6 background were injected subcutaneously (s.c.) with 5×10^5^ B16-OVA mouse melanoma cells or 3×10^5^ E0771 mouse breast cancer cells at the right flank. Perpendicular tumor diameters were measured using a digital caliper and tumor sizes were calculated as length x width. On day 7, when primary tumors were palpable, animals were randomly assigned to treatment groups. Briefly, CpG or Poly I:C at 25 μg was injected into tumor tissues in a volume of 50 μl of PBS or carrier control PBS) daily for a total of three doses. PD-1 or B7-H1 blocking mAb G4, 10B5 or isotype control IgG (Bio X cell) was administered by an intraperitoneal (i.p.) injection at a dose of 200 μg/mouse on day 7 then every three days after for a total of 5 doses. Intratumoral injection of mouse IL-12 (0.5 μg, Peprotech) or anti-mouse IL-12 antibody (10 μg, clone C17.8, BioLegend) were performed daily on days 8-10 post tumor inoculation followed with T cell analysis on day 11. Tumor growth was evaluated every 2 to 3 days until days 35-40 when all mice were euthanized. In compliance with animal care guidelines, mice were euthanatized when either primary or secondary tumors reached 300 mm^2^.

### Flow cytometry analysis of intracellular cytokine production

For cytokine production by tumor-reactive CD11a^high^ PD-1^+^ CD8^+^ T cells [9, 27], lymphocytes from tumor tissues or lymphoid organs were cultured with PMA (50 ng/ml)/Ionomycin (500 ng/ml) (Sigma) for 4-5 hours in the presence of Brefadin A (BD GolgiPlug^TM^), washed and then stained with CD8-PE-Cy5 and IFN-γ-FITC or control antibodies according to the manufacturer's instructions (BD Pharmingen). Antibodies for cell surface molecules were purchased from BioLegend, BD Bioscience and eBioscience. Cells were analyzed using a FACSCalibur flow cytometer (BD) and FlowJo version X.10 (Tree Star, Ashland, OR) software.

### Statistical analysis

Tumor growth after different treatments was analyzed by two-way ANOVA. The survival curves were generated by Kaplan-Meier method, and compared with Gehan-Wilcoxon test. A two-tailed Mann-Whitney test was used to assess statistical differences between experimental groups. The changes of PD-1 expression among different groups were analyzed by One-way ANOVA. A p value <0.05 was considered statistically significant. All statistical analyses were performed using GraphPad Prism software 5.0 (GraphPad Software, Inc., San Diego, CA).
